# Employment Sustainability for People with Intellectual Disability: A Systematic Review

**DOI:** 10.1007/s10926-021-10020-9

**Published:** 2021-12-27

**Authors:** Helena Taubner, Magnus Tideman, Carin Staland Nyman

**Affiliations:** 1 School of Health and Welfare, Halmstad University, Box 823, 301 18 Halmstad, Sweden; 2 School of Allied Health, Human Services and Sport, La Trobe University, Melbourne, Australia

**Keywords:** Intellectual disability, Employment, Sustainability, Systematic review

## Abstract

*Purpose* Previous reviews about employment for people with intellectual disability (ID) have left questions about employment sustainability unanswered. Therefore, the aim of this systematic review was to identify and analyse research regarding employment sustainability for people with ID. The research questions were: What research about employment sustainability for people with ID has been published internationally between 2010 and 2020? In the identified studies, how is employment sustainability defined and measured and what are the main findings regarding employment sustainability? *Methods *A systematic review was conducted using eight databases from various disciplines including medical, health, and social sciences. After a selection process, 10 articles remained, and a framework matrix was created to analyse them. An employment sustainability definition was used as an analytical tool. *Results *Ten articles were identified as being about employment sustainability for people with ID. Five of them used qualitative designs and five used quantitative designs. Only four out of ten contained a definition of employment sustainability, and there was an inconsistency in measurement methods. The reported findings in the studied articles were categorised into three types: proportions of long-term employed individuals within the studied population, facilitators and barriers to long-term employment. *Conclusions *There is only a limited amount of research about employment sustainability for people with ID. Nevertheless, a few facilitators and barriers could be identified. There is no consensus about how to define or measure employment sustainability, making comparisons difficult.

## Introduction

For people with intellectual disability (ID), as well as for people in general, having a job is an important aspect of adulthood [1–4]. Employment is beneficial for the individual [5] as well as for the community and society, and the right of persons with disabilities to work “on an equal basis with others” is included in the UN Convention of Rights of People with Disabilities (CRPD, article 27 [6]). Nevertheless, people with disabilities are less likely to be employed than the population in general. The numbers are even more alarming among people with ID [7] despite the known benefits of being employed for this group [5, 8]. Employment has been studied in relation to disabilities in several ways during recent years [9, 10]. But as in most fields, research specifically about people with ID is scarce. As concluded in previous systematic reviews, there are some studies about possibilities for people with ID to *obtain* employment [11] or their *experiences* of working [12]. But what is known about factors influencing employment *sustainability* for people with ID?

In one frequently cited review, Ellenkamp et al. [13] set out to identify and analyse research about obtaining and maintaining work for employees with ID. Based on 26 identified articles (published between 1993 and 2013), they concluded that “very few studies have focused on work environment-related factors that can enhance competitive work for people with intellectual disabilities” but that “relevant work environment-related factors for obtaining and maintaining work in competitive employment include supporting the employers by paying specific attention to: employer’s decisions, job content, integration and work culture and job coaches” (p. 56). However, they [13] did not separate research about obtaining employment from research about maintaining employment. In addition, Ellenkamp et al. [13] referred to several studies that claim to be about sustainability, but only one of them (Reid and Bray [14]) actually is. Overall, Ellenkamp et al. [13] did not answer questions specifically about maintaining employment but concluded that “evidence for factors that are associated with sustainable work participation are limited” (p. 67).

In another review, Cheng et al. [15] focused on interventions within “open employment settings”, which they defined as “work in the regular workforce for which workers with disability receive wages and conditions of employment commensurate with workers without disability” (p. 318). Although these employments are within the regular workforce, the job seekers with disability may receive specialist support from outside of the workplace (“open employment services”). This “open employment” arrangement is similar to the internationally known framework of Supported Employment (SE), in which the ‘place-train-maintain’ model is frequently adopted [8]. The idea is to first assist people with disabilities to obtain regular employment and then to keep supporting them throughout their training with the goal of creating sustainable employment for the person. SE is well established in the USA as well as in Sweden [16] and the other Scandinavian countries. There is a body of research that has evaluated SE as a method for obtaining employment [8], i.e., the ‘place’ phase of the programme, but very little research about the ‘train’ phase and even less about the ‘sustain’ phase. It is worth noting that the term “supported employment” has previously been used in Australia to label work in sheltered workshops, which contrasts with the international meaning of the term [15].

Like Ellenkamp et al. [13], Cheng et al. [15] did not separate obtaining employment from maintaining employment in their scoping review covering the period between 2001 and 2015. Another similarity between Cheng et al. [15] and Ellenkamp et al. [13] is that they both conclude that within the small existing body of research there is a focus on individual factors influencing the possibilities to obtain and maintain employment for people with ID and that social factors are even less studied.

When conducting a review about securing and maintaining employment among youth with disabilities, with a special focus on the role of gender, Lindsay et al. [9] included research about various types of disabilities. ID was not mentioned explicitly, but similar diagnoses such as learning disabilities were included in the analysis. Out of 48 studied articles, only 6 were about maintaining employment over time. Lindsay et al. [9] concluded that there are “several gender-related barriers and facilitators to maintaining employment including social supports and gender role expectations” (p. 232) but also that their findings “may highlight the importance of exploring specific types of disability” (p. 248) when studying employment.

Burke et al. [17] also reviewed the literature about hiring and retaining people with disabilities, and similar to Lindsay et al. [9] they included all kinds of disabilities (including ID). However, they did not use inclusion criteria or search terms concerning work retention, nor did they report such factors specifically. In addition, they only included research published in the US and not internationally. Thus, overall Burke et al. [17] did not answer questions about international research concerning employment sustainability for people with ID.

Two more recently published reviews [18, 19] addressed the issue of employment specifically for people with ID. Both Nevala et al. [19] and Garrels and Sigstad [18] analysed research published between 1990 and 2019, but neither of them included issues of sustainability. In addition, Garrels and Sigstad [18] only included studies from the Nordic countries, which means that a wider international perspective is once again left out.

To conclude, previous reviews about employment for people with ID still leave questions about employment sustainability unanswered. Factors influencing employment sustainability—both facilitators and barriers—need to be identified in order to assist people with ID not only to obtain, but also to maintain employment. Therefore, the objective of this systematic review was to identify and analyse research about employment sustainability for people with ID. In contrast to Cheng et al. [15], who exclusively studied interventions, this review focuses on regular work life settings within which the employee earns regular wages and works alongside colleagues without disabilities but may receive various types of support. The research questions (RQs) are:

RQ 1. What research about employment sustainability for people with intellectual disability has been published internationally between 2010 and 2020?

RQ 2. In the identified studies,

- how is employment sustainability defined and measured?
- what are the main findings regarding employment sustainability?

## Method

### Database Search

Two domains of search terms were identified (see Table 1)—one domain concerned relevant disability terms, such as “intellectual disability”, and the other concerned sustainable employment, such as “maintaining work”. Terms in both domains were carefully selected by identifying which terms have previously been used in related research. The authors are experienced within disability research in general and in research about ID in particular, and this was an asset when identifying relevant search terms. For instance, the phrase “special education needs” was included based on the authors’ previous knowledge. Two university librarians assisted in the search process, and scholars from relevant research areas were consulted about the terms in the employment domain.

**Table 1:** Search domains and terms

Combination of domains (AND)					
Disability domain			Employment domain		
intellectual OR learning OR developmental	NEAR/1	disabilit* OR handicap* OR impairment* OR disorder* OR difficult*	maintain* OR sustain* OR remain* OR retain* OR retention OR tenure OR keep*	NEAR/2	work* OR employ* OR job* OR occupation* OR vocation* OR career*
OR”special education needs” OR”mental retardation”					

Test searches were performed to ensure accuracy. The actual data collection was then performed in three steps, in which the two domains were first used separately and then combined. The data collection was conducted in early 2021, resulting in 2020 being the latest complete year included. The labour market is constantly influenced by, for example, financial politics and developments, and over time the welfare systems may change. Therefore, research may become outdated over time. In this review, going back to 2010 was deemed sufficient to identify research that was still relevant, and thus the timespan was set to 2010–2020.

**Fig. 1:**
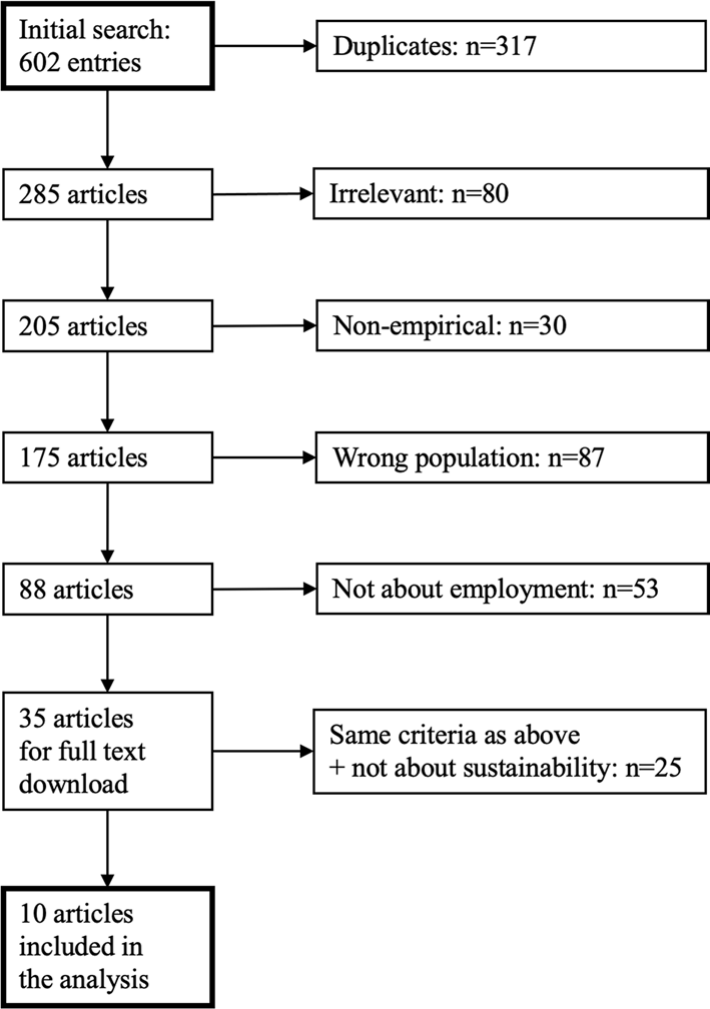
Selection process, PRISMA flowchart

Eight databases were chosen based on the expertise of the authors and of the librarians, with the intent to include databases from the medical, health, and social sciences in order to mirror the multidisciplinarity of the disability research field. The selections of databases in previous reviews (e.g. Ellenkamp et al. [13] and Cheng et al. [15]) were also consulted. The databases used to identify eligible peer-reviewed articles were as follows, with the number of hits in each database presented in parentheses: ABI (9), Cinahl (72), ERIC (45), Medline (68), Psycinfo (124), Scopus (129), Sociological abstracts (4), and Web of Science (111). The data collection search was performed on February 24, 2021, with December 31, 2020, as the latest included publication date, and this resulted in a total of 602 entries. Duplicates (n = 317) were removed automatically using the EndNote software, leaving 285 unique entries (see Fig. 1).

### Study Selection

The 285 entries were scanned manually using the systematic review software Rayyan [20]. Each entry consisted of meta-data (e.g. title, authors, journal) and an abstract. The selection process is shown as a PRISMA flowchart [21] in Fig. 1. Inclusion criteria, which all needed to be fulfilled for an article to be eligible, were that

- the research was original (empirical), peer-reviewed, and reported in English
- the studied population included people with ID (referred to as listed in the disability search domain, see Table 1)
- sustainable employment was actually investigated
- the employees earned regular wages and worked alongside colleagues without disabilities, but could receive various types of support
- the employments had the potential of being long-lasting (e.g. not summer jobs)

Exclusion criteria, of which fulfilling one was enough for an article to be excluded, were that

- the studied groups had other diagnoses (e.g. autism) without co-occurring ID
- other groups (e.g. family members or staff) were the ones who were employed
- the studies were based on interventions

During the initial selection phase, decisions were made based on titles and abstracts using Rayyan [20]. This initial selection (see Fig. 1) resulted in articles being excluded due to being irrelevant (n = 80, e.g. medical studies about working memory, which were identified during the search because they contained phrases like “keeping work memory”), being non-empirical (n = 30, e.g. book reviews, debates, or editorials), being about the wrong population (n = 87, e.g. autism with no co-occurring ID or parents being the ones working), and not being about employment (n = 53, e.g. only mentioning work as part of everyday life but without studying it).

The remaining 35 articles were downloaded in full text format for further examination. In addition to the inclusion and exclusion criteria used during the initial selection process (see above), articles were now excluded if they were not about employment *sustainability*. The total number of excluded articles during the full text phase was 25 (see Fig. 1).

Throughout the entire selections process, the first author prepared preliminary decisions in Rayyan [20] and then discussed them with the second and third authors until consensus was reached. Rayyan provided a feasible tool to keep track of which articles to include, exclude, or discuss further. Eventually, based on consensus between all three authors, 10 articles were eligible to be included in the subsequent analysis.

### Analysis

To analyse our findings we drew on an employment sustainability definition suggested by Kellard et al. [22], that sustainability is “the maintenance of a stable or upward employment trajectory in the longer term”, preferably over a period of at least one year (p. 20). As such, employment sustainability is determined by “personal characteristics and circumstances and by labour market opportunities’ (p. 20). This means that both individual, interpersonal, and societal factors, such as labour markets policies or welfare system structures [23], are influential for whether a person is sustainably employed. In addition, Kellard et al. [22] argue that because “employment sustainability is a multifaceted, composite term” it “does not lend itself to measurement by means of a single indicator” (p. 126). Instead, employment sustainability needs to be measured by taking both “duration” and “trajectories and progress” into account [22]. Thus, employment sustainability not only concerns being long-term employed, but also experiencing a stable or positive career development. Questions asked during the analysis were:

- Do the identified definitions and measurements of employment sustainability concern duration or trajectories?
- Do the studies’ follow-up periods last more than 1 year?
- How do the findings of the studied articles relate to the dimensions of personal characteristics, circumstances, and labour market opportunities?

The analysis was performed using a framework matrix [24] created with data from each of the 10 articles. During the analysis process, the matrix’s columns comprised meta-data (authors, publication year, title, journal, and country), methodological information (qualitative/quantitative, data collection method, studied population, and number of participants with ID), and facts related to the research questions (aim, definition and measurement of sustainability, and main findings). The matrix was then filled with facts and quotations from each article, and the quotations were then categorised using the definition of Kellard et al. [22]. This procedure was performed by the first author, and when any uncertainties emerged all three authors made decisions together. For instance, methods used to measure sustainability were not always presented explicitly, and in such cases all three authors discussed how to interpret what was actually measured. Consensus was reached on all occasions. No additional information was sought about the articles to clarify uncertainties. Because the number of included articles were limited (n = 10), and the characteristics of the articles were diverse, comparisons between them were impossible. The findings are therefore reported descriptively without any meta-analyses or deeper comparisons.

## Results

### Description of Studies (RQ 1)

Ten articles [25–34] met the inclusion criteria. Half of them [25–28, 31] had quantitative designs, and the other half [29, 30, 32–34] had qualitative designs. They were from seven different countries and they were published between 2013 and 2020. Each article was published in a unique journal. The characteristics of each study, including their main findings, are presented in Table 2.

**Table 2:** Characteristics of the included articles (RQ 1 and RQ 2b)

Author, country, and reference	Aim	Method	Participants^a^ with ID	Findings regarding employment sustainability
Båtevik, Norway [25]	How the qualifications and selected life course changes in early adulthood influence the probability of maintaining employment in adult life	Quantitative Longitudinal Two measurements, 8 years apart Structured questionnaire	253 persons former SEN students	65.2% were long-term employed The probability of long-term employment is more than twice as large for men compared to women The probability of long-term employment is significantly higher among women who have achieved a formal upper secondary education qualification compared to those who have not. Such formal qualifications are important for men as well. Nevertheless, contrary to women, for men holding a driver’s license seems to be just as important as formal educational qualifications for maintaining employment
Chan et al., USA [26]	1) Investigated the proportions of adults with ASD with co-occurring ID who were successful in sustaining community employment over an 18-month period of time 2) Examined how individual characteristics, family factors, and contextual influences contributed to sustained employment	Quantitative Longitudinal Two measurements, 18 months apart Oral surveys with mothers	406 persons with ASD and co-occurring ID	14.3% achieved sustained employment More independent daily living skills, a higher family income, a larger maternal social network, an inclusive school environment in early childhood, and currently living in an area with a larger population size were associated with significantly greater odds of sustaining employment Follow-up analyses suggested that managing personal care is particularly important for employment [sustainability]
Heron et al., USA [27]	Increase our understanding of local employer practices and barriers regarding the recruitment, hiring, and retention of individuals with IDD	Quantitative Survey at an employer conference	No participants with IDD (but 44 employers of persons with IDD)	A lack of advancement potential and attitude of customers was mostly reported to be ‘somewhat of a challenge’ to the retention of employees with IDD
Holwerda et al., Netherlands [28]	Investigate which factors predict sustainable work participation, including both finding and maintaining employment, among young adults with mild ID	Quantitative Part of a cohort study. Register data and self-reported data	735 persons with mild ID	15.0% worked for at least 6 months Expectations regarding future work level and living situation predicted /…/ maintaining employment for at least 6 months Living situation refers to living with parents or living independently, i.e. not living in residential institutions No substantial differences between predictors for finding employment and maintaining employment
Lindstrom et al., USA [29]	Examined career development and early employment experiences among four young adults with intellectual and developmental disabilities Research question: What supports are provided to maintain employment?	Qualitative Multiple-method, multiple case-study, longitudinal design (4 years) Interviews combined with documents and observations. Three interviews with each person with ID	4 persons with ID	Over the next 6 to 8 years, participants maintained steady patterns of part-time employment Once entering the labor market, a combination of ongoing training and supervision, flexible employers, and supportive co-workers created the support system needed for job maintenance and stability over time
Meltzer et al., Australia [30]	Examines the systemic barriers they [people with ID] report to finding and maintaining work in open employment	Qualitative In-depth, semi-structured interviews	51 persons with ID	Barriers to maintaining work in open employment include being subjected to experiences of stigma and discrimination from employers, supervisors, and colleagues, varying from feeling undervalued in their job to experiencing both subtle and overt discrimination
Park and Park, South Korea [31]	Analyzed factors affecting the acquisition and retention of employment among individuals with intellectual disabilities (ID)	Quantitative Survey conducted as interviews	398 persons with ID	Being married, absence of basic living security assistance, greater householder income level, family support, and manufacturing industry work were associated with increased job retention
Petner-Arrey et al., Canada [32]	To better understand the experiences of people with IDD in gaining and keeping productivity roles	Qualitative Semi-structured interviews	74 persons with IDD (out of which 21 were represented by someone else)	The results demonstrate the importance of parents and other members of social and family networks relative to connecting with work options and sustaining work over time, especially through continued advocacy and investment
Teindl et al., Canada [33]	To explore how visibility of a disability influences employment for adults with developmental disabilities	Qualitative Semi-structured interviews	3 persons with ID	Easier to find, but not keep, a job with an invisible disability [such as ID]
Vlachou et al., Greece [34]	Exploring the experiences of workers with ID who received supported employment services in Greece	Qualitative Semi-structured interviews	9 persons with ID	Maintaining employment and keeping a job offering security were the primary work-related goals of the participants. More than half of the participants expressed a willingness to remain in the job they had already acquired

^a^ID = intellectual disability, SEN = special education needs (including persons with ID), IDD = intellectual and developmental disabilities, ASD = austism spectrum disorder

### Definitions and Measurements of Employment Sustainability (RQ 2a)

Only four of the ten included articles explicitly stated how employment sustainability was defined (see Table 3). Båtevik [25] reported two measurement points with 8 years in between and defined sustainability as being employed at both of these points. Chan et al. [26] also applied a definition based on being employed at two points in time, but the timespan was considerably shorter (18 months). Holwerda et al. [28] defined employment sustainability as being employed for six consecutive months. And finally, Park and Park [31] applied a timespan of 1 month in their definition. The other six articles did not define employment sustainability at all.

**Table 3:** Definitions and measurements of employment sustainability (RQ 2a)

Article	Definition of employment sustainability	Measurement of employment sustainability
Båtevik [25]	Holding a permanent job in both in 2007 and 2015	The dependent variable, long-term employment, refers to former SEN students registered in permanent work in 2015 as well as in 2007. Permanent work includes, in this case, different combinations of work and disability benefits
Chan et al. [26]	Working for pay independently and/or with support in the community greater than 10 h/week in total at two measurements [18 months apart]	The mother of the person with ID reports that they are employed at both measurements
Heron et al. [27]	–	[Employers] rated their challenges with the retention (e.g., lack of advancement potential, the actual cost of accommodating a disability, attitudes of customers) of employees with IDD (1 = not a challenge, 2 = somewhat a challenge, 3 = major challenge)
Holwerda et al. [28]	Working for at least six consecutive months	Twelve quarterly measurements during the follow-up period, which per individual ranged from 1 year and 3 months to 2 years and 9 months
Lindstrom et al. [29]	–	Participants’ accounts from three interviews over 4 years and triangulation with other types of data
Meltzer et al. [30]	–	Participants’ accounts from one interview
Park and Park [31]	Having a job for at least one month	Self-reported information. Job retention was coded as the month of the survey year minus the year and month of employment
Petner-Arrey et al. [32]	–	Participants’ accounts from one interview (unclear which questions were asked about sustainability)
Teindl et al. [33]	–	Participants’ accounts from one interview about their experience of seeking, finding, and maintaining meaningful employment
Vlachou et al. [34]	–	Participants’ accounts from one interview about facilitators and barriers for maintaining employment

The quantitative articles [25–28, 31] considered employment sustainability as being employed at certain consecutive points in time (see Table 3), and they collected data in two main ways: registers and surveys. Holwerda et al. [28] used register data to assess whether their participants were employed at certain points in time over a timespan of 6 months. The other four articles were based on survey data. Chan et al. [26] collected survey data orally from mothers of persons with ID, while Park and Park [31] performed an oral survey with persons with ID. As long as the participants were school-aged, Båtevik [25] used registration forms that were filled out by teachers, and when the participants had become adults they replied to structured questionnaires themselves (or in a few cases with assistance). And finally, Heron et al. [27] conducted a survey at an employer conference with employers of people with ID.

All five qualitative articles [29, 30, 32–34] were based on semi-structured interviews (see Table 2). Employment sustainability was thus measured in terms of accounts given by the participants during the interviews. Meltzer et al. [30], Petner-Arrey et al. [32], Teindl et al. [33], and Vlachou et al. [34] conducted interviews on one single occasion, while Lindstrom et al. [29] conducted three interviews with each participant with ID over a period of 4 years. Because their interviews were in part retrospective, Lindstrom et al. [29] included a timespan of between 6 and 8 years. In addition to interviewing persons with ID, Lindstrom et al. [29] conducted observations, document analyses, and interviews with family members and staff.

### Reported Findings About Employment Sustainability (RQ 2b)

The reported findings about employment sustainability (see Table 2) may be categorised into (1) proportions of long-term employed individuals within the studied population, (2) facilitators and (3) barriers. First, the proportions of long-term employed individuals within the studied populations varied considerably. Båtevik [25] reported that 65.2% of the 253 participants were employed at both measurement points (which were 8 years apart). Chan et al. [26] reported sustained employment over 18 months in 14.3% of the 406 studied persons with ID and co-occurring autism. And finally, Holwerda et al. [28] reported that 15.0% of the participants with mild ID were employed during the 6 months of the study. The large difference between these numbers may partly be explained by the fact that Båtevik [25] used the term “special education needs”, which includes other groups alongside people with ID.

Second, facilitators for employment sustainability were, on an individual level, having a formal upper secondary education or a driver’s license [25], having more independent daily living skills, including personal hygiene [26], having expectations regarding future work level [28], and being a man [25]. Regarding the social situation surrounding the person with ID, facilitating factors were living in a family with a higher income or a larger maternal social network [26], living with parents or independently (i.e. not in residential institutions) [28], receiving ongoing training and supervision at work, having flexible employers and supportive co-workers [29], being married, having a supportive family or working in manufacturing industries [31], and experiencing continuing advocacy and investment from parents and other social networks [32]. On a societal level, facilitating factors were having been in an inclusive school environment in early childhood or currently living in an area with a larger population size [26] and living without basic living security assistance or in a household with greater income level [31]. Worth noting is that Holwerda et al. [28] did not find any “substantial differences between predictors for finding work and maintaining employment” (p. 1986).

Third, the reported barriers were lack of advancement potential or a negative attitude from customers [27] and being subject to experiences of stigma and discrimination from employers, supervisors, and colleagues [30]. These barriers concern the work environment of the person with ID. On an individual level, Teindl et al. [33] reported that having an invisible disability (such as ID) made it more difficult to keep a job over time (compared to people with more visible disabilities), and Båtevik [25] reported that the probability of long-term employment was more than twice as large for men compared to women. Having an invisible disability or being a woman are therefore barriers to long-term employment.

### Analysis Based on Kellard et al.’s Definition

Kellard et al. [22] state that employment sustainability is “the maintenance of a stable or upward employment trajectory in the longer term” (page 20), and they argue that it needs to be measured by taking both “duration” and “trajectories and progress” into account (p. 126).

Regarding “stable or upward employment trajectory”, none of the explicit definitions in the included articles contained anything about employment trajectories. Instead, the four explicit definitions were based on “duration” rather than “trajectory and progress”. The only finding related to employment trajectory was reported by Heron et al. [27], who reported that employers of people with ID reported lack of advancement potential to be somewhat of a challenge for job retention.

The next part of the definition concerns the longer-term perspective. Kellard et al. [22] argue that studies about employment sustainability should include a timespan no shorter than 1 year, not least because it is necessary to include an entire year in order to capture seasonal differences in labour market trends. In this review, the timespan was very different among the studied articles and varied from 1 month [31] to 8 years [25, 29]. Only three [25, 26, 29] of the articles included a period longer than 1 year.

Kellard et al. [22] also state that employment sustainability depends on both “personal characteristics”, “circumstances”, and “labour market opportunities”. The distribution of reported facilitators and barriers across these three dimensions is displayed in Table 4.

**Table 4:** Facilitators and barriers across the three dimensions mentioned by Kellard et al. [22]

	”Personal characteristics” [22]	”Circumstances” [22]	“Labour market opportunities” [22] or other societal factors
Facilitators	Having a formal upper secondary education or a driver’s license [25] Having more independent daily living skills, including personal hygiene [26] Having expectations regarding future work level [28] Being a man [25]	Living in a family with a higher income or a larger maternal social network [26] Living with parents or independently (i.e. not in residential institutions) [28] Receiving ongoing training and supervision at work, having flexible employers, and supporting co-workers [29] Being married, having a supportive family, or working in manufacturing industries [31] Experiencing continuing advocacy and investment from parents and other social networks [32]	Having been in an inclusive school environment in early childhood or currently living in an area with a larger population size [26] Living without basic living security assistance or in a household with greater income level [31]
Barriers	Having an invisible disability (such as ID) [33] Being a woman [25]	Lack of advancement potential or a negative attitude from customers [27] Being subjected to experiences of stigma and discrimination from employers, supervisors, and colleagues [30]	–

Some “personal characteristics” of the employees with ID were reported in the studied articles in terms of both barriers and facilitators. The social situation in which the person with ID lives and works corresponds to their “circumstances”. On the societal level, Båtevik [25] included social background as one of the covariates, but concluded that “there seems to be no correlation between the social background and long-term employment” (page 162) within the studied group. No barriers on the societal level, including “labour market opportunities”, were reported, while the reported facilitators on the societal level were having been in an inclusive school environment in early childhood or currently living in an area with a larger population size [26] and living without basic living security assistance or in a household with greater income level [31]. These factors exceeded “labour market opportunities”. For instance, inclusive schools or household income levels go beyond “labour market opportunities”. We therefore argue that the definition by Kellard et al. [22] is too narrow on the societal level, and when presenting the facilitators and barriers in Table 4 “labour market opportunities” is combined with other societal factors.

## Discussion

The findings of this systematic review show that there is a very limited amount of research about employment sustainability for people with ID, especially regarding societal facilitators (reported by two studies) and barriers (not reported in any of the studied articles). We expected to find reported barriers concerning, for example, labour market demands regarding education or lack of support systems aimed at employers to be of importance, but surprisingly no such factors were presented in the studied articles. Thus, this review confirms the findings of Cheng et al. [15] and Ellenkamp et al. [13] who concluded that there is a need for further research about the societal factors that influence employment for people with ID.

The findings also highlight the lack of consensus about how to define and measure employment sustainability. There is a trade-off between on the one hand having too short a duration and on the other hand having measurement points too far separated and thus losing the possibility to study the trajectory between those points. Båtevik [25] acknowledged the latter problem when stating that although their study spanned over a long period of time (8 years) nothing was known about the participants’ trajectories between the two measurements. We agree with Båtevik [25] that a large timespan with a limited number of measurements causes an uncertainty about the trajectory between the measurement points. In addition, we agree with Kellard et al. [22] in their opinion that the duration should be at least 1 year in order to ensure the possibility to disregard seasonal shifts and instabilities over shorter periods of time (e.g. sick leaves or vacation periods). Thus, there needs to be a balance between duration and trajectory when defining sustainability, as stated by Kellard et al. [22].

The studied articles varied in definitions and measurements, but they were also diverse in several other ways. Definitions and terms concerning disabilities, support systems, and work life vary considerably between countries and scientific traditions, thus leading to great difficulty in comparing the studies. There is, for instance, some confusion about the terms “open employment” and “supported employment” in relation to their use in Australia (see Cheng et al. [15]). In addition, there is a difference between welfare systems in the respective countries, leading to a variation in access to support for people with ID [23]. As a result, labour market opportunities may vary considerably between countries, and comparing studies internationally is thus difficult.

The greatest difficulty in comparing studies is, however, the great variation in diagnoses within the studied material. In most cases [28–31, 33, 34], the diagnosis of ID is used as an inclusion criterion (but it is rarely stated how it was defined or controlled). In some articles [27, 32], however, the term Intellectual and Developmental Disabilities (IDD) is used instead, and in one study [26] ID co-occurs with autism spectrum disorder (ASD). Finally, Båtevik [25] uses the term “special education needs”, which includes people with ID but without separating them from other sub-groups. In addition, there are different traditions and procedures regarding diagnoses in different countries, meaning that the studied populations are not comparable. Thus, we conclude that there is uncertainty as to whether the articles study the same groups.

Because the body of publications is so small and heterogenous, there are only limited possibilities to base decisions on general research findings when designing support systems. Thus, it is therefore likely that the inconsistent use of definitions and terms leads to missed opportunities to assist people with ID in obtaining and maintaining employment.

### Future Research

We agree with Holwerda et al. [28] that “factors influencing finding work by individuals with ID may differ from factors influencing maintaining employment” and that it is therefore “important to take sustainability of employment into account as well” (p. 1983). On the other hand, Holwerda et al. [28] did not find substantial differences between predictors for finding employment and for maintaining employment. More research is therefore urgently needed about factors influencing employment sustainability and their relation to factors influencing obtaining employment among people with ID.

Vlachou et al. [34] emphasised that all of the participants in their study stated that maintaining employment was a primary goal because it provided security and economic independence. This highlights the importance of continued research about factors that facilitate or hinder employment sustainability for people with ID because of its relevance to the persons themselves (see also, for example, Salt et al. [3] and Jahoda et al. [8]). Drawing on the findings of Lindsay et al. [9] as well as those of Båtevik [25], issues of gender should be taken into account when doing further research.

We suggest more multi-method research within this field. On one hand, qualitative methods are valid for capturing retrospective accounts about trajectories and the experiences of being long-term employed. On the other hand, quantitative methods are valid for performing statistical analyses involving larger populations. To study the complexity of employment sustainability—“a multifaceted, composite term that does not lend itself to measurement by means of a single indicator” [22] (p. 126)—both of these approaches are needed.

Last but not least, further research is needed to define employment sustainability for people with ID. The present lack of consensus regarding definitions and measurements results in difficulties in comparing the limited body of publications, which in turn may reduce the chances to create support systems aimed at ensuring employment sustainability for people with ID.

### Limitations

Research outcomes are always dependent on the researchers’ preconceptions and decisions made during the research process. It is therefore important to reflect on potential bias [21]. The authors of this review are experienced researchers within the disability field, and our pre-existing knowledge about ID strongly influenced the search strategy. For instance, the term “special education needs” was included based on our previous knowledge of Scandinavian disability research. There might have been a geographical bias within the included material such that research from other parts of the world than those familiar to the authors was missed. However, articles from all over the world (including Scandinavian and North American countries, but also, for example, the United Arab Emirates, South Korea, Israel, Australia, and Slovenia) *were* identified in the initial data, which shows that the possible risk of geographical bias was minimized. In addition, the expertise of the assisting librarians was a great asset in creating the search strategy as their knowledge about terms and databases widened the perspective of the authors. Publications in other languages than English were excluded, however, meaning that there may be research in other languages that was not identified.

## Conclusion

This review set out to identify and analyse previous research about employment sustainability for people with ID, and three research questions were posed. As few as ten articles met the inclusion criteria, and these originated in seven different countries and half were quantitative studies and half were qualitative studies.

This review highlights a lack of a consensus definition of sustainability and an inconsistency in measurements. Only four of the studied articles explicitly stated how they defined employment sustainability, and all four of these definitions were about duration rather than progress or trajectory. The timespans within these definitions varied between 1 month and 8 years. The five quantitative articles used register or survey data to measure employment at different points in time, while the five qualitative articles used semi-structured retrospective interviews. Because both definitions and measurements were so diverse, no meta-analyses or deeper comparisons were possible.

The main findings presented in the analysed articles may be categorised into (1) proportions of long-term employed individuals within the studied population, (2) facilitators of long-term employment, and (3) barriers to long-term employment. The facilitators and barriers were distributed across the dimensions of “personal characteristics”, “circumstances”, and “labour market opportunities” [22], showing an apparent lack of reported factors, especially barriers, on a societal level. Thus, in line with previous reviews (see Cheng et al. [15] and Ellenkamp et al. [13]), this review shows that very little research is available about societal factors related to employment sustainability for people with ID.

To conclude, questions about employment sustainability for people with ID are still not satisfyingly answered.

## Data Availability

Not applicable.
